# 

**Published:** 1902-09-27

**Authors:** 


### NOTICE TO CORRESPONDENTS.

All MSS., Letters, Books for Review, and other matters intended for the Editor should be addressed The Editor, "The Hospital," The Hospital Building, 28 & 29 Southampton Street, Strand, W.C.

The Editor cannot undertake to return rejected MS., even when accompanied by stamped directed envelope.

**Notice to Advertisers and Subscribers.**

All Advertisements, Orders for Copies of The Hospital, and Business Communications should be addressed to **THE MANAGER, (Not to the Editor)**, The Hospital, 28 & 29 Southampton Street, Strand, London, W.C.

The Cost of Subscription, post free, is as follows.—Per week, 2½d.; per quarter, 2s. 8d.; per half-year, 5s. 3d.; per annum, 10s. 6d. Foreign:—Per annum, 15s. 2d., payable, in all cases, in advance.

**NOTICE.—Vol. XXXI. of "The Hospital" (October 1901, to March 1902), in handsome cloth binding is now on sale at the office of this Journal, price, 7s. 6d. Cases for binding the Numbers contained in this Volume 1s. 6d. Index to Vol. XXXI., 2½d., post free.**

**The Monthly Part for October will be ready on October 1. Price 6d., by post 8d.**

### BOOKS RECEIVED.

Macmillan and Co.

"Reports from the Cancer Research Laboratories: The Middlesex Hospital."

Baillière, Tindall and Cox.

"Suggested Standards of Purity for Goods and Drugs." By C. G. Moor, M.A., F.I.C., F.C.S.

Adlard and Son.

"Report of the Tuberculosis Committee of the Medico-Psychological Association."

J. B. Lippincott Company.

"International Clinics." Edited by Henry W. Cattell, A.M., M.D.

Simpkin, Marshall, Hamilton, Kent and Co.

"Notes on Optics for Students of Ophthalmology." By A. S. Percival, M.A., M.B.

Aberdeen University Press.

"A Contribution to the Ætiology of Cancer." By Alex. Theodore Brand, M.D., C.M.

John Bale, Sons and Danielsson.

"Friedreich's Paralysis." By T. Duncan Greenlees, M.B., F.R.S.E., and G. Carrington Purvis, M.D., B.Sc.

P. Blakiston's Son and Co.

"Massage and the Original Swedish Movements." By Kurre W Ostrom.

### THE STUDENT OF MEDICINE.

### APPOINTMENTS.

**T. Ruell Atkinson**, M.D.Durh., M.R C.S.Eng., appointed Public Vaccinator for the Third District of the Romford Union. **H. M. Cade**, M.R.C.S., L.R.C.P.Lond., appointed District Medical Officer of the Grantham Union. **William Duncan**, M.B., C.M.Glasg., appointed Certifying Factory Surgeon for the Clay Cross District of Derbyshire. **Henry B. Ellerton**, M.R.C.S.Eng., L.R.C.P.Lond., appointed Senior Assistant Medical Officer to the Leavesden Asylum. **Hunter Finlay**, M.D., appointed Health Officer for the Town and District of Davyhurst in the Ularing District, Western Australia. **Duncan Fletcher**, L.R.C.P.&S.Edin., L.F.P.&S.Glasg., D.P.H., appointed Chief District Medical Officer of Health for the Harris District of the County of Inverness. **L. P. Gordon**, M.R.C.S., L.R.C.P.Lond., appointed Medical Officer and Public Vaccinator for the Brailsford District of the Ashbourne Union. **Lucy Gullett**, M.B., Ch.M.Syd., appointed Resident Medical Officer of the Children's Hospital, Brisbane, Queensland. **Ronald Campbell Macfie**, M.B., M.S.Aberd., appointed Medical Superintendent to the Dundee Sanatorium. **R. W. H. Meredith**, M.R.C.S., L.R.C.P.Lond., appointed District and Workhouse Medical Officer of the Wellington Union, Somerset. **G. F. E. Morgan**, L.R.C.P.Lond., M.R.C.S.Eng., D.P.H., appointed District Medical Officer to the Hartlepool Union. **Martin R. Morrissey**, L.R.C.P.&S.Edin., appointed Certifying Factory Surgeon for the Ballymore Eustace District of the County of Kildare. **R. A. Mossman**, L.R.C.P.&S.Edin., appointed District Medical Officer of the Ripon Union. **P. F. O'Hagan**, L.R.C.P.&S.Edin., appointed Certifying Factory Surgeon for the Croydon District of Surrey. **J. B. Page**, M.B.Lond., appointed District Medical Officer of the Sudbury Union. **W. Paisley**, M.B., B.Ch., B.A.O., R.U.I., appointed Medical Officer to the Cape Government Railways, Queenstown, Cape Colony. **A. J. Partridge**, M.B., B.S.Vict., appointed Medical Officer of the Training Ship *Exmouth* of the Metropolitan Asylum District. **Pryce Peacock**, M.D.Brux., L.R.C.P.&S.Irel., appointed Certifying Factory Surgeon for the Castleknock District of the County of Dublin. **Harry A. Robinson**, M.D., B.Ch.Vict., appointed Senior Assistant Medical Officer to the Darenth Asylum. **Henry Naunton Robson**, M.R.C.S.Eng., L.R.C.P.Lond., appointed House Surgeon to the General Hospital, Stroud, Gloucestershire. **Digby S. Sandiland**, M.R.C.S., L.R.C.P.Lond., appointed Senior House Surgeon to the West Ham and East London Hospital, E. **James H. Sequeira**, M.D.Lond., M.R.C.P.Lond., F.R.C.S.-Eng., appointed Assistant Physician to the Skin Department of the London Hospital. **Edgar Wm. Sharp**, M.B., Ch.B.Glasg., appointed Resident Medical Officer to the Halifax Workhouse Infirmary. **W. H. Steele**, M.B., C.M.Edin., appointed District Medical Officer of the Newton Abbot Union. **Wm. E. Taylor**, M.B., Ch.B., appointed Resident Physician to the Aberdeen City Hospital. **J. A. Valentine**, M.D., B.Ch., B.A.O.Univ.Dub.Trin.-Coll., appointed House Surgeon to the National Eye and Ear Infirmary, Dublin. **J. Humphry Williams**, M.D., C.M.Edin., reappointed Medical Officer of Health to the Flint Town Council.

**"The Hospital" Institutional Library.**

"Hospitals and Asylums of the World." 4 Vols., with a Portfolio of Plans, complete £12 12s.
"Burdett's Hospitals and Charities, 1902." 5s.
"Cottage Hospitals, General, Fever, and Convalescent." 10s. 6d.
"Hospital Expenditure: The Commissariat." 2s. 6d.
"A Legal Handbook for the Use of Hospital Authorities." 2s. 6d.
"The Cottage Hospital Case Book or Register of Patients." 10s. and 12s. 6d.
"The Furnishing and Appliances of a Cottage Hospital." 6d.
All these are published by the Scientific Press, Ltd., and may be obtained through any bookseller, or direct from the publishers, 28 and 29 Southampton Street, Strand, London, W.C.

### SCRAPS AND GLEANINGS.

The new Campbell Hospital at Perth is designed to provide accommodation for twenty patients. The amount of the contracts is in all £3,418.

The report of the Prison Commissioners for the year ending March 31st, 1902, shows that the daily average of prisoners detained in the local prisons was 16,267, the highest since 1885.

### Medical and Administrative Appointments.

**MEDICAL DEPARTMENT OF THE NAVY,**
ADMIRALTY, Northumberland Avenue, W.C.
10th September, 1902.

An EXAMINATION of CANDIDATES for entry into the Medical Department of the Royal Navy will be held in NOVEMBER next (probably on the 17th and following days) at Examination Hall, Thames Embankment.

The number of Commissions offered will be announced later.

The forms to be filled up by candidates will be supplied on application to this Department.

H. F. NORBURY,

Director-General.

(2836)

**HAMLET OF MILE END OLD TOWN.**

ASSISTANT MEDICAL OFFICER WANTED.

The Guardians of the Poor of the Hamlet of Mile End Old Town require the services of a Gentleman, registered under the Medical Act, 1858, &c., and duly qualified to practice Medicine and Surgery, as RESIDENT ASSISTANT MEDICAL OFFICER at the Workhouse Infirmary, Barncroft Road, Mile End, E., also as ASSISTANT MEDICAL OFFICER to the Workhouse.

The salary, which will be an inclusive one, has, subject to the approval of the Local Government Board, been fixed at £150 per annum, with Board, Lodging, and Laundry, and an allowance of 2d. per day in lieu of Beer.

Candidates, unmarried and not exceeding 40 years of age, are invited to attend at the Guardians' Offices, Barncroft Road, Mile End Road, London, E., on THURSDAY, the 9th day of October, at half-past three o'clock in the afternoon, and bring with them a written application on foolscap stating particulars of present and past services.

The appointment will be subject to the approval of the Local Government Board, and also subject to the provisions of the Poor Law Officers' Superannuation Act 1896.

The gentleman appointed will afterwards have to produce originals of all testimonials, diplomas, &c.

Canvassing the Guardians is prohibited, and no travelling or other expenses will be paid. By order,

Guardians' Offices, WILLIAM THACKER,
Barncroft Road, Mile End, E., Clerk to the Guardians.
September 11th, 1902. (3140)

**WARDMASTER REQUIRED BY THE GOVERNMENT**
OF HONG KONG IN GOVERNMENT MEDICAL DEPARTMENT.

The engagement will be in the first instance for three years with salary at the rate of £110 a year. If the services of the officer selected are satisfactory to the Government, he will at the end of three years be placed on the pensionable establishment of the Colony with salary at the rate of £130 a year, rising after three years to £150 a year. For purposes of local payment the salary will be converted into dollars at a rate of exchange fixed annually by the Government and based on the average exchange value of the dollar during the preceding twelve months.

Free second class passage to Hong Kong and return passage to this country if services are not retained after three years. Candidates must be single and not more than 30 years of age, and must have had previous hospital training. Preference will be given to a member of the Royal Army Medical Corps.

Free furnished quarters for a single man and free uniform will be provided.

The selected candidate will be required to pass a strict medical examination as to his fitness to serve in a tropical climate.

Applications, stating age and experience, accompanied with copies (not originals) of recent testimonials, with the names and addresses of references of whom inquiries can be made, should be sent to the Crown Agents for the Colonies, Downing Street, London, S.W., by the 30th September, 1902.

Applications should bear the following reference on the top left-hand corner $\frac{m}{1557}$ (3127)

Demy 8vo., with two Plates, cloth gilt, 12s. 6d. net.

**MEDICAL HISTORY FROM THE EARLIEST TIMES.**

By E. T. WITHINGTON, M.A., M.B. Oxon.

The Scientific Press, Ltd., 28 & 29 Southampton Street, Strand, London, W.C.

